# PLA Renewable Bio Polymer Based Solid-State Gamma Radiation Detector-Dosimeter for Biomedical and Nuclear Industry Applications

**DOI:** 10.3390/s22218265

**Published:** 2022-10-28

**Authors:** Wen Jiang, David DiPrete, Rusi P. Taleyarkhan

**Affiliations:** 1 School of Nuclear Engineering, Purdue University, W. Lafayette, IN 47907, USA; 2 Savannah River National Laboratory, Aiken, SC 29808, USA

**Keywords:** polylactic acid (PLA), gamma dosimetry, mass loss ratio (MLR), porosity

## Abstract

Polylactic acid (PLA) as a “green,” renewable corn-soy based polymer resin was assessed as a novel solid-state detector for rapid-turnaround gamma radiation dosimetry in the 1–100 kGy range–of significant interest in biomedical and general nuclear industry applications. Co-60 was used as the source of gamma photons. It was found that PLA resin responds well in terms of rheology and porosity metrics with an absorbed gamma dose (Dg). In this work, rheological changes were ascertained via measuring the differential mass loss ratio (MLR) of irradiated PLA placed within PTFE-framed (40 mm × 20 mm × 0.77 mm) cavities bearing ~0.9 g of PLA resin and pressed for 12–16 min in a controlled force hot press under ~6.6 kN loading and platens heated to 227 °C for the low Dg range: 0–11 kGy; and to 193 °C for the extended Dg range: 11–120 kGy. MLR varied quadratically from 0.05 to ~0.2 (1σ ~ 0.007) in the 0–11 kGy experiments, and from 0.05 to ~0.5 (1σ ~0.01) in the 0–120 kGy experiments. Rheological changes from gamma irradiation were modeled and simultaneously correlated with void-pocket formations, which increase with Dg. A single PLA resin bead (~0.04 g) was compressed 5 min at 216 °C in 0–16 kGy experiments, and compressed 2 min at 232 °C in the 16–110 kGy experiments, to form sturdy ~100 µm thick wafers in the same press. Aggregate coupon porosity was then readily measurable with conventional optical microscope imaging and analyzed with standard image processing; this provided complementary data to MLR. Average porosity vs. dose varied quadratically from ~0 to ~15% in the 0–16 kGy range and from ~0 to ~18% over the 16–114 kGy range. These results provide evidence for utilizing “green”/renewable (under $0.01) PLA resin beads for rapid and accurate (+/−5–10%) gamma dosimetry over a wide 0–120 kGy range, using simple to deploy mass and void measuring techniques using common laboratory equipment.

## 1. Introduction

Gamma radiation is omnipresent in daily life. From safety and utility considerations, gamma dosimetry is utilized worldwide in a wide range of industries and disciplines. For the past over 80+ y, ionizing radiation monitoring technology has remained largely the same [1,2,3,4]–relying primarily on sensor technologies that require monitoring for the tell-tale charge buildup in ionized gases/solids (e.g., fission chambers using highly enriched uranium and compensated ion chambers), or monitoring of light flashes from scintillation or thermoluminescence. Complex and bulky radiation spectrometers can cost in the $M range (as may be deployed at high powered accelerator-driven spallation sources or research reactor facilities and require skilled scientific staff), down to a range of $1–10 K for portable survey meters, and even at as low as $10/detector for commonly used personnel dosimeters (e.g., TLDs).

The need for gamma dose measurements can span a large dose range. At the low level, the dose can be as low as ~10^−8^ Gy (1 μRad) for cosmic background levels. In the intermediate range of ~1–10 kGy, it could apply, for example, to food irradiation and packaging sterilization [5]. At the higher levels, to 100 kGy and higher, it is applied in diverse fields, such as in medicine [6,7,8,9,10], high power nuclear reactors, and accelerator-driven systems where the exposure dose rates may exceed 10^4^ kGy/h (10^9^ R/h).

As is evident, it would be a desirable outcome if one could develop and demonstrate a potentially transformational advance in radiation and associated dose monitoring technology, resulting in a novel, nonpowered solid state, ultra-lightweight-scalable [e.g., ~1 g (~2 mm size) detector], affordable (<$0.1/unit), corn-soy polylactic acid (PLA) biodegradable, environmentally friendly, easy-to-use, general purpose gamma-beta-alpha-fission-neutron monitor that is readily deployable (especially in extreme, e.g., 1000–100,000 R/h) radiation fields for use in ensuring facility safety and operations across the DOE nuclear infrastructure, and for enabling deployment over a wide range of ambient temperatures. Previously, we have published results of scoping efforts to develop a PLA-based solid-state radiation detector (PLAD) based on monitoring irradiated PLA specimens for morphological changes using FTIR- and relative viscosity (RV)-based techniques [11,12]. In this paper, we present advances in PLAD technology that permit gamma detection-dosimetry using significantly simplified techniques based on the underlying physics of PLA rheological changes that nicely correlate with gamma radiation.

What is so compelling for proposing PLAD, which is based on monitoring for tell-tale damage caused by ionizing radiation in materials? It is well-known that ionizing radiation interaction will produce atom dislocations, electron transitions and other effects in virtually all materials. For example, neutron irradiation of steel walls can result in embrittlement. However, such dislocations and property changes (e.g., melting temperature, color or viscosity) are *not* readily discernible for monitoring in real time using commonly available devices and techniques. What is needed is a material that responds reasonably well, even in harsh nuclear environments, to varied forms of ionizing radiation to produce well-correlated property changes to simple physical properties (e.g., density, relative viscosity, hardness, Hf, molecular weight, color changes) that are amenable to rapid-fire and cost-effective measurement using common laboratory equipment. **Specifically, this paper presents results pertaining to gamma radiation dosimetry enablement based on PLA polymer resins for application in the 1–100 kGy dose range. That is, to develop a PLA-based dosimeter, we will refer to it as PLAD** to offer a potentially viable and novel breakthrough alternative to present-day gamma radiation monitors.

### Brief Introduction to PLA and Industrial (Medical, Food Packaging, Coatings-Adhesion, etc.)

**Figure 1:**
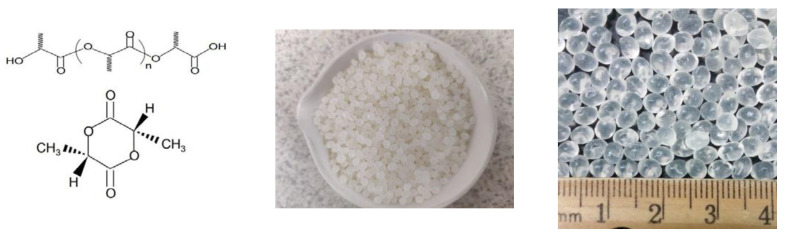
PLA molecular structure, ~3 mm resin beads in cup and closeup with scale–PLA resin supplied by Natureworks, LLC.

Polylactic acid (PLA) is a “green,” corn-soy based biopolymer and has been widely used in medical applications such as implants, surgical sutures, drug formulations and deliveries [6,7,8,9,10], food packaging industries [6,7,10], and also as an adhesive [11,13,14,15]. Its NFPA (National Fire Protection Association) rating is “0 1 0” for safety, flammability and reactivity. Figure 1 provides salient information on the molecular formula and physical characteristics of PLA resin beads. 

## 2. Problem Formulation-Research Framework/Objectives on Advancing PLAD Technology for Gamma Dosimetry in Medical and Other Nuclear Applications

Scoping studies (in-house at Purdue University and elsewhere) have revealed that gamma irradiation of PLA can significantly alter morphology and physical properties [12]. The morphological changes alter both the polymer in terms of MW and the associated strength when subject to ionizing radiation with and without crosslinking. It was hypothesized that the mechanical effects should also alter the rheological flow properties of the PLA resin itself, such that it would flow and exhibit macroscopic microstructural (void/pore formation) variations in direct correlation to the absorbed dose– both features acting as metrics for absorbed gamma dose and hence result in PLAD as a rapid-readout gamma dosimeter. 

The application of PLA in medical instruments potentially opens up its possibility for internal dosimetry, yet the dose levels for radiotherapy thus far are generally no more than 100 Gy [16,17]. However, potentials still lie in the dosimetry in X-ray facilities, where the dose rate could reach 480 Gy/h [18], and in food packaging and sterilization industries, where the dose applied could reach 10 kGy [5] and above for chem-bio agent defeat. Significantly higher levels of gamma dose must be considered when deciding on the choice of polymer materials for radiation-sterilized products [19]–for the most part through 100 kGy and even toward 4000 kGy depending on the specific polymer chosen. 

Consequently, it was decided to focus the present study on evaluating PLAD for functionality as a novel, low-cost and near real-time dosimeter for dose monitoring in the 1–100 kGy dose range; this decision was also made out of practical considerations since this dose range could be conveniently accommodated via constrained access to Purdue University’s Co-60 GammaCell^TM^ irradiator.

The challenge problem-objective then was to derive, research and demonstrate methods for measuring rheological and voiding metrics using techniques that are simple and that use widely available equipment.

Experiments and protocols were developed to study two physical effects of gamma dose (Dg) effects on PLA resin on: 

(1) Changes in heated resin deformation and mass loss when subject to mechanical compression, and

(2) Monitoring the average void fraction (i.e., porosity) in irradiated PLA resin. 

The next section provides details of the experimentation and the results obtained.

## 3. PLAD Gamma Dosimeter-Related Experimentation

This section discusses the experimental setup and related equipment used for assessing PLAD for gamma dosimetry in the 0–100 kGy range. 

### 3.1. PLAD Resin Type Used for Studies

Due to its ready availability and experience in use as an adhesive, NatureWorks^®^ Ingeo™ biopolymer resin 4043D was used in this study, which is a semi-crystalline polymer. Some typical properties are listed in Table 1 [20]. Figure 1 provides a view of the colorless transparent resin beads supplied by NatureWorks, LLC. 

### 3.2. Gamma Irradiation Source for Studies

**Figure 2:**
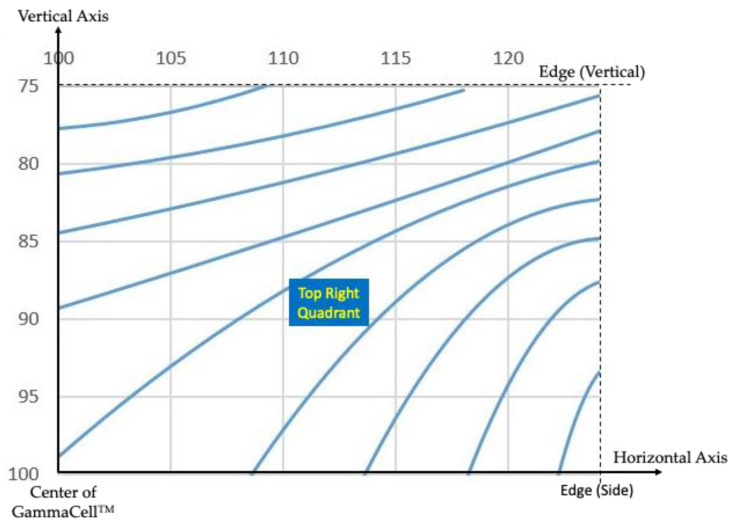
Relative Gamma Dose Map for Nordion GammaCell^TM^ (Data from Ref. [23]).

Purdue University’s Nordion GammaCell 220^TM^ Co-60 irradiator [22] was used to perform γ photon irradiation of the PLA resin samples. The average dose in the unit was initially calibrated [23] with Fricke dosimetry [24] in 1993 and the dose rates extending to the time of usage were evaluated based on the decay of the Co-60 source. The accuracy of the estimated dose rate is ±0.56% at the 95% confidence limits. By the summer of 2021, when irradiation was performed, the dose rates were, in general, on the order of 2 kGy/day. The dose map provided by the manufacturer is reproduced in Figure 2.

The dose evaluated by Fricke dosimetry was converted to the actual dose absorbed by PLA. Nevertheless, the converted dose has little difference to which Fricke dosimetry evaluates, since the Mass Absorption Coefficient (provided by the National Institute of Standards and Technology database [24]) of ferrous sulfate, the main component of standard Fricke dosimetry, is very close to that of PLA exposed to 1.25 MeV photons, the average energy of 1.17 and 1.33 MeV for Co-60 photons (0.02955 cm^2^/g for ferrous sulfate and 0.02816 cm^2^/g for PLA).

**Figure 3:**
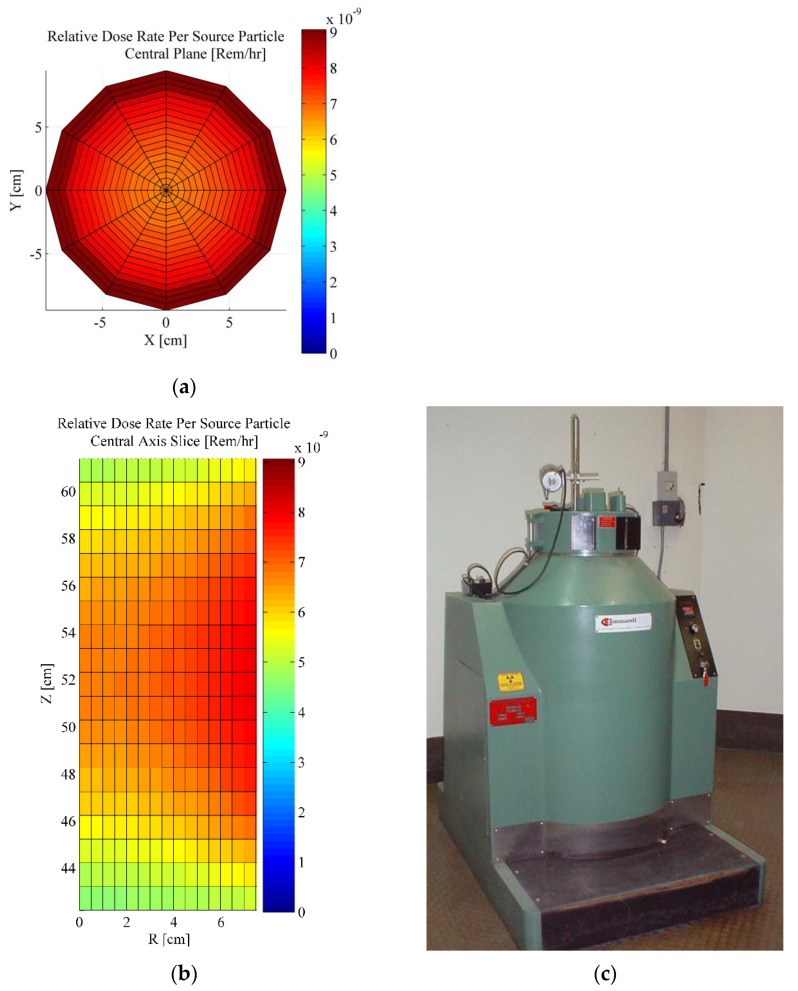
MCNP code model results [11] of dose rate variations in Purdue University’s Gammacell Irradiator: (**a**) Mid section, axial; (**b**) R-Z slice, radial; (**c**) Irradiator [22].

A MCNP code simulation [11] was also built characterizing the irradiator core for its spatial radiation dose rate profile, results of which were used to guide for the positioning of samples used for this current study. Figure 3 provides the results of dose variation with radial (from the centerline) and axial locations within the irradiator. PLA resins were irradiated in the presence of room air. The maximum dose received was 114.4 kGy (2021). 

From both evaluations, it was found that the dose rate at the wall was about 20% higher than at the center. During irradiation, the samples were kept at the center area of the chamber, and the bottles containing the resins were shaken from time to time to unify.

### 3.3. Apparatus for Mass Flow-Based Gamma Dosimetry

#### 3.3.1. MLR-Based Sample Preparation

**Figure 4:**
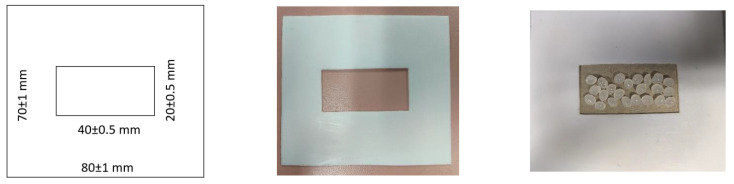
Schematic and pictorials of PTFE frames with and without PLA resin PTFE (Polytetrafluoroethylene sheets provided by McMaster Carr^TM^, melting range 327–342 °C, relative density 2.14–2.19 g/cm^3^ at 20 °C; the thickness of the sheet is 0.77 ± 0.01 mm.

In order to evaluate and quantify the rheological changes with gamma irradiation dose, it was decided to configure a system for operations, as shown schematically in Figure 4.

The system comprises a cavity within a thin polytetrafluoroethylene (PTFE) enclosure frame into which a mass (m_o_) of PLA resin material (with or without irradiation) is placed evenly within the cavity space–both of which are shown in Figure 4. The area dimension of the cavity was chosen from practical considerations to align with that of a single flattened PLA resin bead (commonly sold as feedstock worldwide by NatureWorks, LLC.)–such that, upon compression, a measurable quantity of the molten PLA material can egress out of the cavity. PTFE was chosen as the frame material due to its non-wetting property and ability to withstand adhesion to molten PLA, thereby permitting the controlled flow of PLA under pressure and heat. This coupon is then compressed in a hot press, during which the molten PLA is allowed to flow out of the cavity (i.e., create a mass release “m_r_”) to differing levels, depending on irradiation-based changes in overall relative viscosity. An extra technical issue needed attention. Under hot press compression, molten PLA adhesion to upper–lower steel surfaces must be avoided. To achieve this requirement, during the hot-press compression stage, a thin sacrificial release liner was required between the frame and the steel sheets on either side. Through trial-error, a commonly used food industry packaging material sold in grocery stores-parchment paper from Big Chef^®^ was found to be suitable. The parchment paper specifications are: thickness: 0.045 ± 0.005 mm, maximum temperature for safe usage: 218 °C; non-wettable to PLA melt and water–in laboratory tests the parchment paper exhibited 106.5 ± 6.7 degrees contact angle with 0.5 µL deionized-filtered water drop. 

The resultant mass loss ratio (MLR) metric is then derived as

MLR = m_r_/m_o_. (1)

where, m_r_ is the PLA mass amount that is released from the cavity region, and m_o_ is the original PLA mass within the cavity, respectively.

#### 3.3.2. Hot Press for Molten (Irradiated) PLA Flow Studies

**Figure 5:**
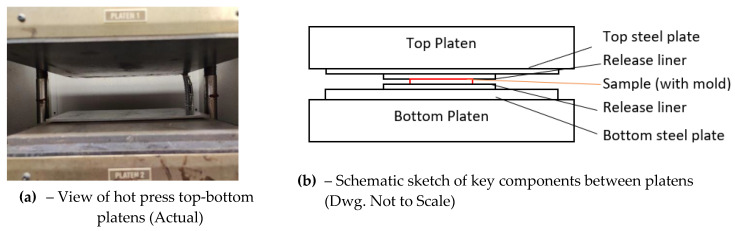
Pictorial and schematic of the hot press and key components for MLR studies.

A state-of-the-art high-fidelity apparatus [Carver Model AutoFour/30-1H^TM^ manufactured by Wabash MPI, located in Wabash, IN, USA] was deployed for this study, as shown in Figure 5a. This hot press enables controlled, radially uniform compression under temperature-to provide up to 294,000 N clamping force using 4 vertical columns with two 0.38 m × 0.38 m electrically heated steel-platens. The pre-programmed platen temperatures were varied from ~177 °C (350 °F) up to 232 °C (450 °F) in this study. This press permitted programmable application of desired loads, duration of compression and pre-determined hot platen surface temperatures (with minimal radial temperature variation–which we also verified and characterized). We used a K-type thermocouple positioned at various distances from the centerline to map out the temperature profile. It was found that measured temperature varies from the set temperature with increased distance from the centerline–varying from a low of ~0.33 °C (~0.4%) to ~1.67 °C (~1.5%) at the centerline to about 1.17 °C (~1.5%) to 3.3 °C (~3%) at the edges. However, for the present study, the PLA bearing sample is only 0.04 m × 0.02 m, and is deliberately positioned at the center of the press; the temperature variations are expected to be <0.1 °C over the dimension of the coupon samples. 

As mentioned earlier, our studies have shown that PLA can serve as a hot-melt adhesive for joining a vast array of materials, including steel, thereby potentially contaminating the platen surfaces in the case of leakage past the release liner. As an added precaution, a 0.3 m × 0.3 m × 0.013 m (12″ × 12″ × 0.5″) steel plate was placed between the bottom platen and the sample, while a 0.15 m × 0.3 m × 0.005 m (6″ × 12″ × 0.2″) steel plate was placed between the top platen and the sample, as shown in Figure 5b.

### 3.4. Underlying Logic for Choice of MLR Testing Parameters

This section discusses the logic and reasoning behind adopting the test parameters.

#### 3.4.1. PLA Resin Amount

As mentioned earlier, the cavity size was chosen to accommodate ~1 g of PLA resin. To optimize MLR studies, the issue of the PTFE (0.77 mm thick, 20 mm × 40 mm) mold expansion under heating conditions also needed to be taken into account. Based on the coefficient of thermal expansion of PTFE provided in the datasheet [25], the volumetric expansion of the Teflon^®^ PTFE mold is ~4% when heated from room temperature to 193 °C and ~5% when heated to 227 °C. Since 0.8 g of PLA is enough to fill an expanded PTFE mold when heated to 193–227 °C, an excess 0.1 g was deemed appropriate for deriving significant MLR values; therefore, 0.9 g was eventually chosen as the mass amount of PLA.

#### 3.4.2. Hot Press Compression Loading Level

**Figure 6:**
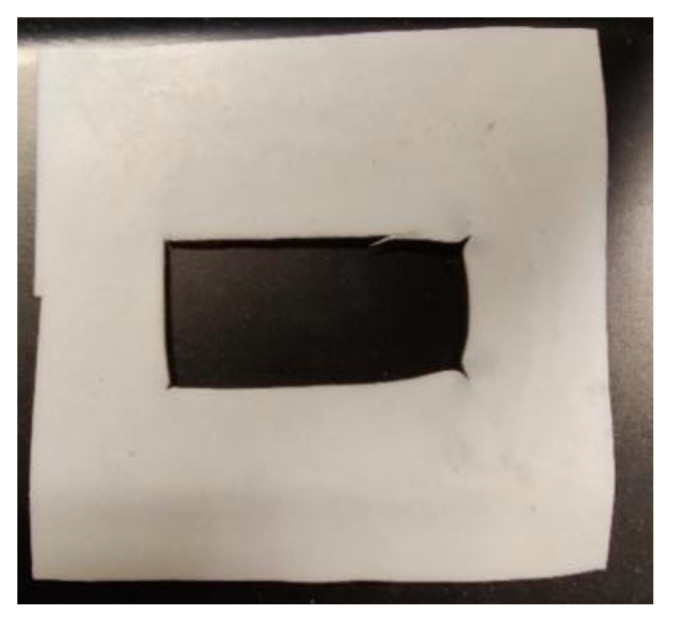
Damaged PTFE mold compressed under 44,480 N at 227 °C.

The minimum stated force that the Carver^TM^ Press can apply is 4448 N (1000 lbF). While the load for MLR studies is preferred to be kept as low as possible for the purpose of allowing significant amounts of PLA to escape, it was also found that the press could not maintain stable loading significantly below 6672 N (1500 lbF). Higher forces could also be applied, but if too high a force is applied (e.g., 44,480 N), permanent distortion occurs to the PTFE mold itself, as seen in Figure 6. Consequently, the chosen baseline compression force was set at 6672 N (1500 lbF). 

#### 3.4.3. Temperature Range and Time Duration of Compression for MLR Studies

**Figure 7:**
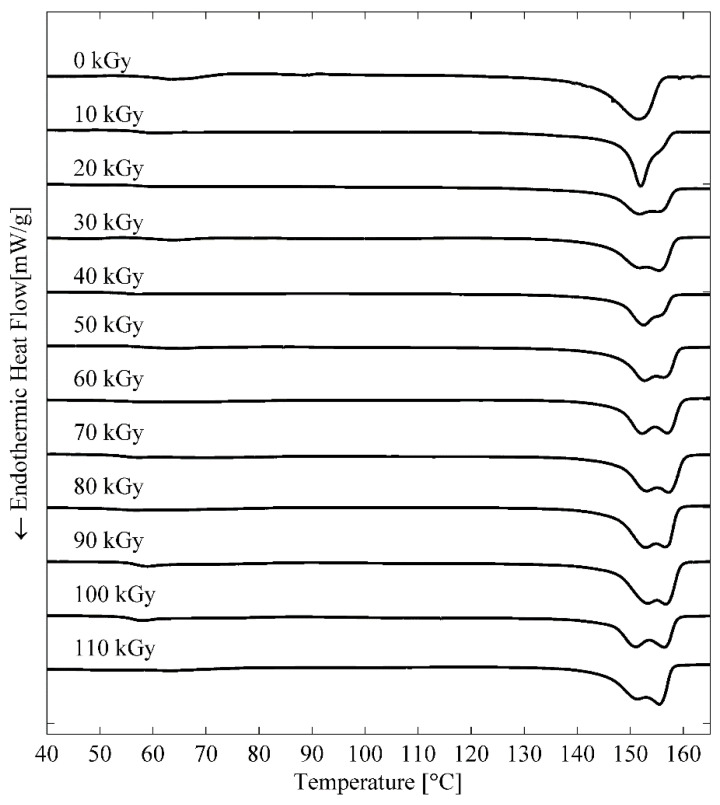
DSC results for irradiated 4043D resin from 10 to 110 kGy [13].

The next decision pertained to the choice of hot press temperature and the associated time duration for compression under temperature. For this, we first review the results of differential scanning calorimetry (DSC). The DSC curves of PLA 4043D resins irradiated with various gamma doses are shown in Figure 7. The curves show that the melting of 4043D resins of various gamma doses starts from ~140 °C and continues until ~160 °C [11,12]. This result agrees with the nominal melting temperature of 4043D (145–160 °C) claimed by NatureWorks^®^ [20]. Another technicality relates to significant PLA decomposition onset at 250 °C as noted from Table 1. 

Based on the above, it was decided that the MLR studies should be conducted with platen temperatures above 140 °C which allow significant material flow, but should not exceed 250 °C. It was also found that the same press temperature and hold time under compression would not be appropriate over the entire 0 to 120 kGy range–i.e., to obtain good resolution at low as well as at high irradiation dose levels. This required finding a suitable combination of MLR-related test parameters for the high (11–120 kGy) range and low (0 to 11 kGy) range, separately. This is discussed below in sequence.

##### MLR Temperature and Hold Time Test Parameters for the 11–120+ kGy Range

**Figure 8:**
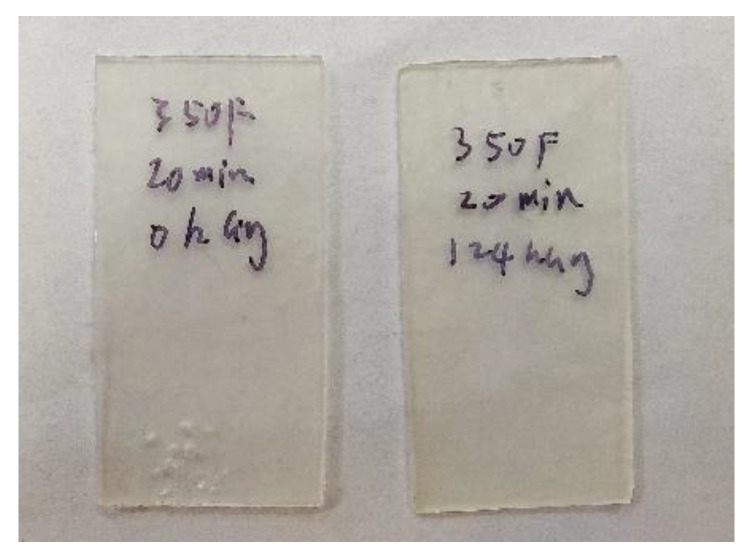
Samples for 0 kGy (**left**) and 124 kGy (**right**, 2014/2015 irradiated) resins tested at 177 °C for 20 min.

During trials, exploration started from 177 °C (350 °F) and an arbitrary (but reasonably long) compression time of 20 min. However, this resulted in only ~12% mass loss for 124 kGy after 20 min of compression (Table 2), which was not deemed to be sufficiently large for allowing good resolution dosimetry from 0 to 100 kGy range. Figure 8 shows the post-compressed PLA sample coupons (i.e., the PLA sample mass remaining within the PTFE mold’s cavity).

**Figure 9:**
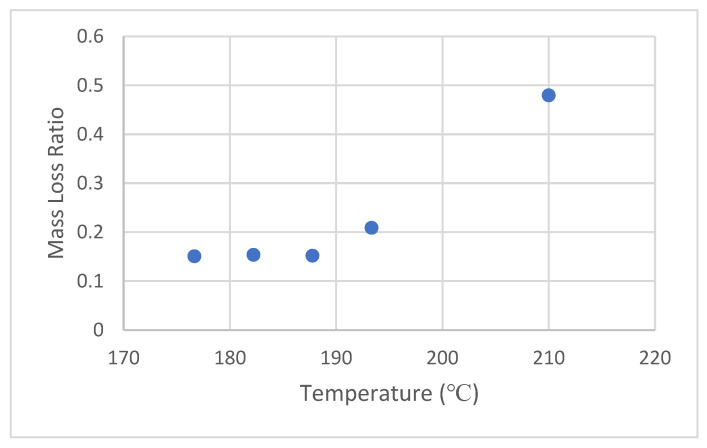
MLR for 124 kGy resins held at various temperatures for 10 min under 6672 N force.

To save time and increase the resolution of dose predictions from 0 to 100 kGy, in lieu of longer compression time, it was first decided instead to assess the effect of temperature on the rheology. Figure 9 shows the results of MLR for 124 kGy irradiated PLA resin, held at various temperatures from 177 °C to 210 °C–all compressed for 10 min duration. The upper end of the temperature scale was chosen to remain compatible with the mass flow rate metric specified by the manufacturer (Table 1)–meant to be the industrial temperature for extrusion.

**Figure 10:**
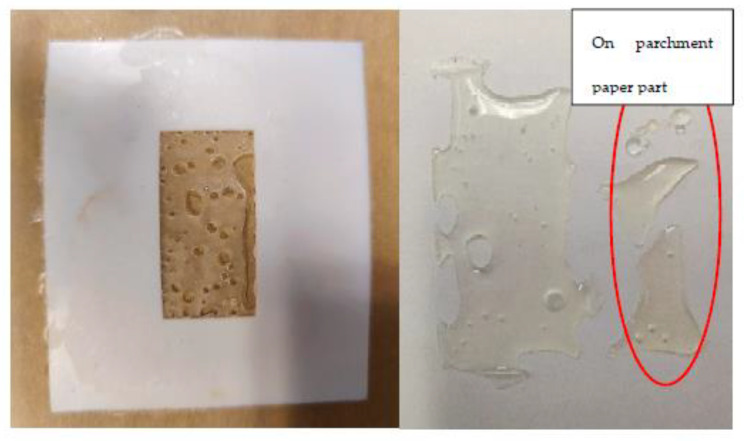
Fragile sample remaining in the mold cavity with holes (**left**) and adhesion to the release liner (**right**).

As seen in Figure 9, the MLR rises rapidly above 190 °C to reach a substantial value of ~0.5 at 210 °C. However, this high level, while exciting, also gave rise to practical issues. Post-compression, the PLA sample became fragile enough so as to make it difficult to handle without shattering and breakage. Furthermore, the PLA resin also starts to attack and adhere to the release liner (see Figure 10), which compromises the accuracy of MLR measurements with high levels of uncertainty. 

In order to avoid the issues discussed above, 193 °C (380 °F) was eventually chosen as the compromise temperature. 

Next, we needed to determine the hold time duration at the temperature. Attempts were then made to find the optimal hold time by determining the MLR for various hold times. 

The inflexion (optimal) time point was deemed to occur at/around 12 min, at which point the MLR was sufficiently high and sample examinations could be conducted without disintegration or adhesion to the release liner. As a side note, at the end of 12 min of compression, an additional 1 min rest time was allowed after releasing the pressure and before retrieving the samples [Note: this protocol allows the top steel plate to slightly cool and reduces the attraction between the plate and the top release liner]. Taken together, we could reach an MLR range of 0.05 to 0.5 for the 11–120 kGy range without significant fragmentation of the sample.

##### MLR Temperature and Hold Time Test Parameters for the 0–11 kGy Range

**Figure 11:**
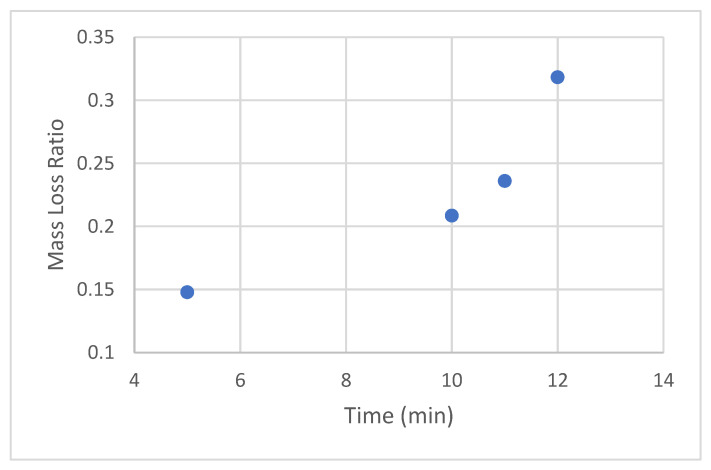
MLR for 124 kGy resins held at 193 °C (380 °F) for various time durations under 6672 N (1500 lbF) [data obtained with single samples at each hold time].

While the combination of hot press compression adequately covered the 11–120 kGy range (shown in Figure 11), the resolution was inadequate for discerning dose effects via MLR for dose levels in the 0–11 kGy range. Considering that the issues pertaining to sample disintegration are more pronounced for higher dose levels, a new optimal set of temperatures and hold times were examined for the 0 to 11 kGy range.

**Figure 12:**
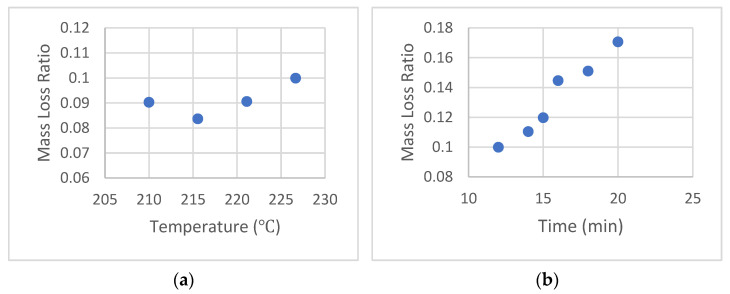
MLR for 9.5 kGy gamma dosed PLA 4043D resins (**a**) MLR-temperature profile held under 6672 N (1500 lbF) for 12 min (**b**) MLR-time profile held under 6672 N (1500 lbF) at 227 °C (440 °F).

As a start, keeping the hold time at 12 min, the MLR was found for an irradiated 9.5 kGy sample at various temperatures from 210 °C to 227 °C (close to the decomposition temperature), for which the results are shown in Figure 12a. Thereafter, the hold time was increased from 12 min through 20 min, and the corresponding results are shown in Figure 12b, which indicates a sharp increase at ~16 min. It was also found that for hold times above 16 min at 227 °C (which is above the recommended temperature of 218 °C for the release liner), the liner started to burn and cause adhesion-related disintegration of the PLA. 

Consequently, for the 0–11 kGy range, the optimal test parameters were set at 227 °C and 16 min hold time. This combination allowed the MLR values to range from 0.05 at 0 kGy to about 0.2 at 11 kGy. 

### 3.5. MLR Experimentation Test Matrix and Procedure 

MLR-related testing was conducted for the test parameters summarized in Table 3.

#### Experimental Procedure

PLA resin beads were irradiated to varying levels from 0 to 114.4 kGy using Purdue’s GammaCell^TM^ irradiator. Taking into account Co-60 decay over time, the gamma doses were evaluated by multiplying the time-averaged dose rate over the irradiation duration by the irradiation time, then converted to the actual dose absorbed by PLA using the method aforementioned in Section 3.2. These irradiated resin beads were then used to prepare coupon samples for placement in the Carver^TM^ Hot Press for set durations of time, depending on the dose range being considered.

**Figure 13:**
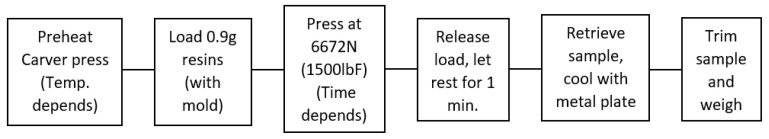
Flow chart showing the protocol for MLR determination.

Figure 13 illustrates in a flowchart the steps taken for testing the irradiated PLA resin in the Carver^TM^ Hot Press. The press was first preheated to the desired temperatures and allowed to stabilize together with the two steel plates used to separate the platens and the sample; then, the PTFE frame was placed between the two steel plates at the centerline of the press. PLA resin beads were weighed and loaded into the PTFE frame. A release liner (parchment paper) covered the top and bottom surfaces, as discussed earlier. It takes ~30 s for the press to reach 6672 N (1500 lbf) after the platens have been closed, before it was held in place for hold and rest times specified in Table 3, respectively. After the platens were raised, the sample was retrieved together with the mold and release liner. The sample was placed on a flat surface, with a metal plate loaded on top until it cooled down. After removing the sample from the mold, the PLA material released out of the central cavity was trimmed, followed by mass measurements. The difference between the original and remaining mass in the mold cavity is the mass loss (ML) and MLR is evaluated per Equation (1). Several samples were prepared for each irradiation dose. 

### 3.6. Porosity-Metrics of Irradiation 

In addition to the MLR, an accompanying metric based on sample porosity was also deemed intriguing for studying irradiation dose-induced changes to the PLA morphology.

**Figure 14:**
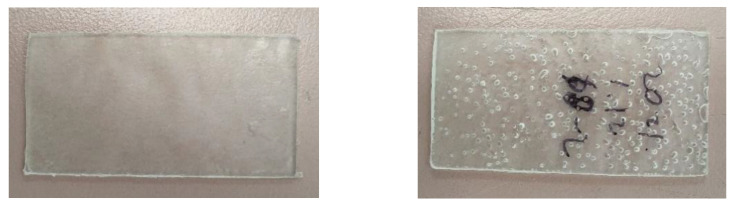
MLR experimentation samples. Left: control (0 kGy)-no visible pores; Right: 56 kGy gamma dose pre-irradiated–showing visible pores.

Interestingly, on a visual basis alone, the post-irradiated PLA resin beads from the GammaCell^TM^ irradiator did not show any signs of void formation, even when viewed under an optical microscope. However, during the aforementioned MLR studies, it was found that gamma irradiation samples, when heated under compression, also gave rise to obvious and significant voiding (porosity) of the PLA material. The degree of voiding-fragmentation increased with irradiation dose. Figure 14 shows the results of the samples for 0 kGy and 56 kGy (adapted to PLA) after subjecting the sample to the Carver^TM^ Hot Press conditions mentioned earlier.

It is uncertain as to what the underlying physical cause of such macroscopic porosity change is, which manifests itself only when subject to heat and compression. A systematic effort was undertaken to derive an associated porosity metric for the absorbed dose.

#### 3.6.1. Effect of Gamma Irradiation Dose (<110 kGy) on Bulk Density

The density change of PLA 4043D resins that underwent various doses of gamma irradiation was first examined by placing 5 g of resin beads of each selected dose in a 50 mL graduate cylinder containing 20 mL of distilled water and dividing the mass of the resin beads (5 g) by the volume change of the water. See Table 4 for a summary.

These results are within measurement uncertainty (for this relatively crude method) when compared with the published nominal density of PLA 4043D (1.24 g/cc) from NatureWorks, LLC. We conclude that irradiation alone through ~110 kGy does not change the bulk density in any significant sense. 

#### 3.6.2. Possible Causes of Porosity in Irradiated PLA “after” Hot Press Compression

Without closer post-irradiation examination (PIE), it is speculated that: (a) radiation-induced chain-scission degradation of the macro-molecules reduces the original strength of the PLA molecular chains linked to the smaller molecules trapped between the chains. The source of the smaller molecules could likely be remnant solvent molecules, additives and water contamination during resin bead manufacturing. These additives (smaller molecules) make it easier for them to evaporate upon heating and lead to porosity; (b) radiolysis-related microbubbles and/or cracks (not visible under an optical microscope) are formed during irradiation; these fault lines then become nuclei for growing bubbles of vapor (water or other additives), which then expand and disrupt the structure when subjected to elevated temperatures. 

In a practical sense, irradiation followed by compression under heat leads to visible and quantifiable porosity, thereby leading to an alternate Dg metric. However, clearly visible to the naked eye, porosity as a metric is not readily quantifiable due to the very significant size distribution of the pores, with sizes ranging from above 10 to 100 microns in effective radius. In order to develop a simple methodology and metric, it was decided to cast irradiated PLA resin beads into thin wafer samples such that the pore size was larger than the thickness of the wafer–this led to the need to press down the irradiated PLA resin beads to a thickness of ≤100 microns.

#### 3.6.3. Unique Protocol for Producing 100 µm thick PLA Samples

At first, the same combination of hot press conditions (i.e., force, temperature and hold time) were assessed as done for deriving the Dg metric using the MLR approach–with the only exception being the mold. Similar setups as for MLR were adopted for producing 100 µm thick PLA samples for the sake of consistency, except that the mold was replaced with surrounding 100 µm aluminum spacer strips, and the samples were replaced with one PLA bead for each dose. Beads used for all measurements had a weight of ~0.040 ± 0.005 g each. The load was also kept at 6228 N (1400 lbf). The same temperature/time conditions (193 °C/12 min) were first applied to determine the porosity of 114 kGy.

**Figure 15:**
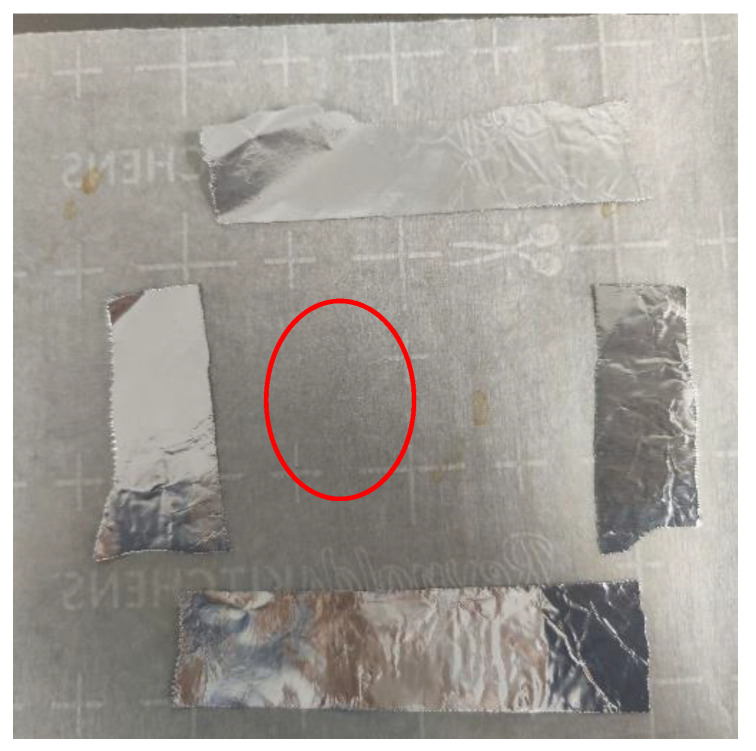
One 114 kGy PLA resin bead compressed under 6228 N, 193 °C/12 min. Remnant PLA resin almost invisible (encircled).

Surprisingly, without the PTFE mold, almost all PLA was lost (adhered to the release liner) with only insignificant disintegrated remnants left on the parchment paper (see Figure 15). 

**Figure 16:**
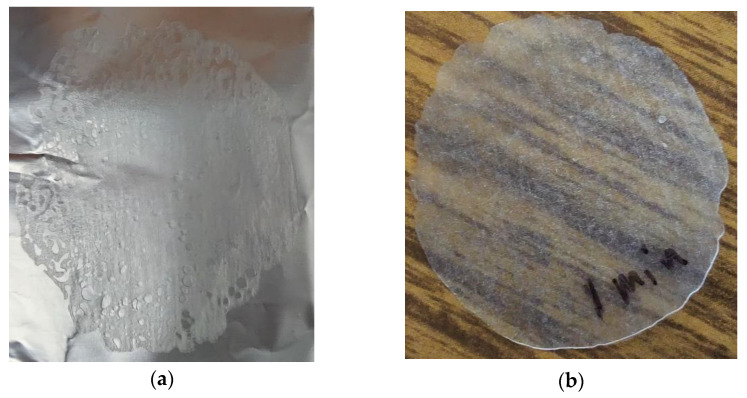
PLA resin beads compressed under 6228 N at 193°C for 1 min. (**a**) 114 kGy–visible pores at the edge; (**b**) 33 kGy–no visible pores.

It was discovered that the hold time was a key factor; upon reducing down to 1 min, a more useful porosity-bearing sample could be derived–albeit, the PLA concentrated at the center while pores merged on the edge with irregular shapes, as seen in Figure 16a. For doses below 33 kGy, no visible pores could be noted, as seen in Figure 16b. 

#### 3.6.4. Protocol for Porosity-Related Dg Metric Derivation

Therefore, it became clear that to derive a porosity-based Dg metric, a different combination of force-temperature-hold times would need to be determined in a process similar to that done for the MLR-based approach. 

Following a similar set of steps as done for the MLR approach through trial-error, the following parameters shown in Table 5 were derived for performing experiments with irradiated PLA to derive a porosity-related Dg metric:

Temperature higher than 232 °C was not selected due to the fact that it is close to the decomposition temperature of PLA (250 °C), and also far exceeds the maximum working temperature of the parchment paper (218 °C)–in which, the thin wafers of larger dose samples (>16 kGy) started to adhere to the parchment paper and was not easy to be retrieved without damaging the samples. The same phenomenon appeared for 114+ kGy samples pressed under 216 °C/2 min conditions, and was even worse, which was why temperatures higher than 216 °C could not be utilized for the high dose range samples. The dose level of 16 kGy effectively became the boundary of the two sets of conditions since pores could barely be found on those samples prepared with 216 °C/2 min conditions, making it the onset of the high dose range and the end point of the low dose range. This was different from that found for the MLR approach.

**Figure 17:**
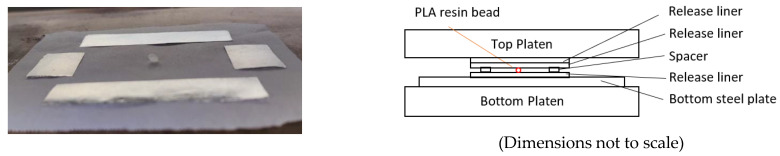
Pictorial and schematic of hot press and key components for porosity measurements.

**Figure 18:**
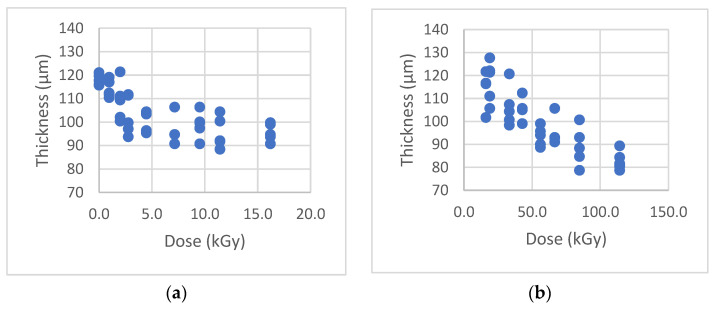
Thickness of the samples for porosity measurements: (**a**) Dose range 0–16 kGy; (**b**) Dose range 16–114 kGy.

Figure 17 (left) shows placement of the PLA bead surrounded by the spacer strips. The spacers were made with 4 layers of Kroger^®^ Heavy Duty Aluminum Foil, for which the total thickness was measured to be 104.0 ± 0.5 µm. For convenience, when preparing the porosity-related wafer samples in the hot press, the top steel plate was not used; since the hold time was shorter (2 or 5 min) compared with that for the MLR approach, every time during the sample loading/unloading process, the temperature of the top steel plate dropped noticeably. An additional release liner was placed above the top release liner instead to avoid direct contact between the top release liner and the top platen of the press, as shown schematically (right) in Figure 17. The thicknesses of the as-derived hot-pressed PLA samples versus the dose are shown in Figure 18.

Interestingly, the thickness of the 0–16 kGy samples (pressed under 232 °C/5 min conditions) stabilized at a mean value of ~100 µm, while the thickness of the 16–114 kGy samples (pressed under 216 °C/2 min conditions) continued to reduce with increasing dose– even below that of the Al-strip spacers. Despite the +/− 10 micron variation in the thickness of the wafers at each dose level, it appears that with further effort, controlled thickness change monitoring itself may also be possible as another simple and straightforward gamma dose metric for the future. Nevertheless, the as-produced wafer samples allowed the examination of pore distributions and sizes with a conventional optical microscope.

**Figure 19:**
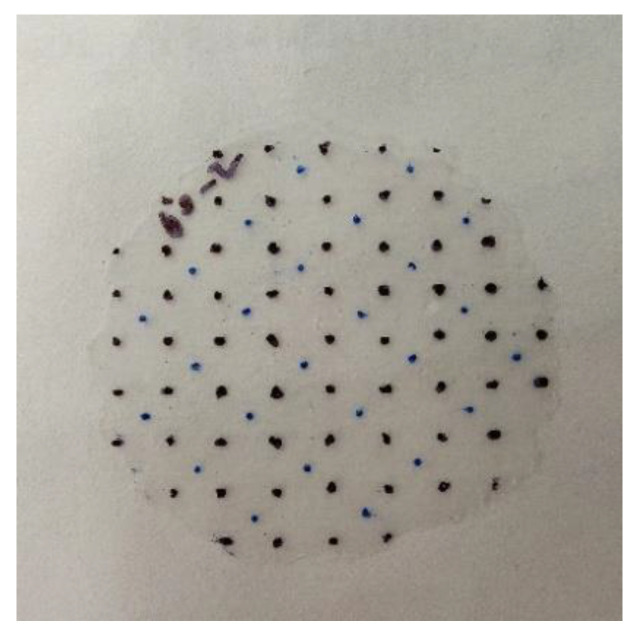
Wafter sample of 56 kGy with ~1 mm × 1 mm grids.

A DCM800^TM^ Microscope was then used for porosity determination. Images were captured through the eyepiece of the microscope using an external digital camera. To determine the porosity consistently, ~1 mm × 1 mm grids were marked on each wafer sample—just enough to be included in the view of the minimum magnification of the microscope (40×). The integral pore areas in every other grid were calculated using the public domain (**ImageJ**) image processing software. An example of a grid is shown in Figure 19. The blue dots are indicators marking the grid locations to be measured for porosity. Two samples were measured at each selected dose.

**Figure 20:**
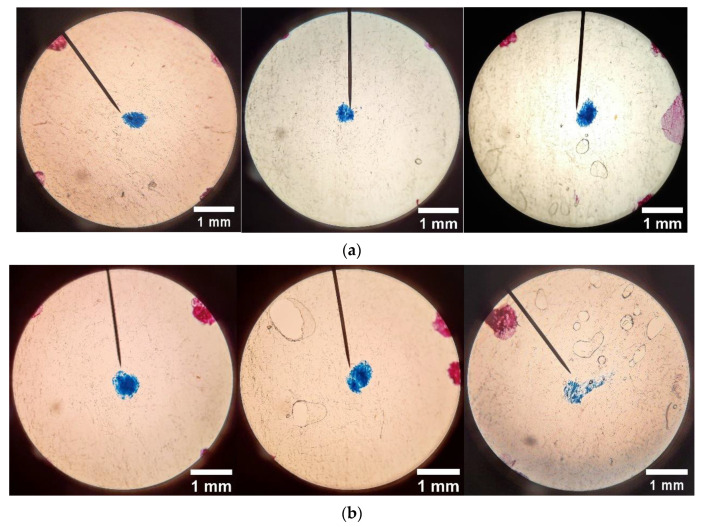

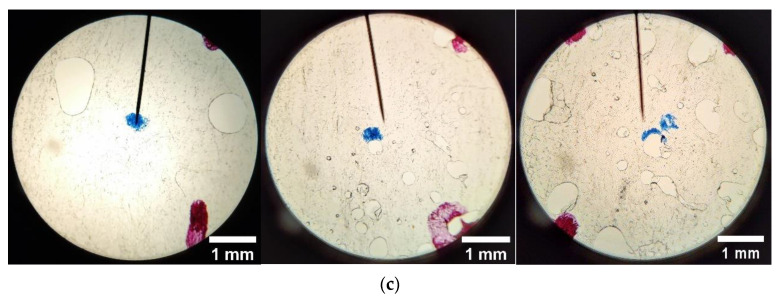
Typical microscopic images (40×) for (**a**) 0 kGy; (**b**) 7.0 kGy; (**c**) 11.2 kGy. Hot Press conditions: 6228 N, 232 °C, 5 min.

**Figure 21:**
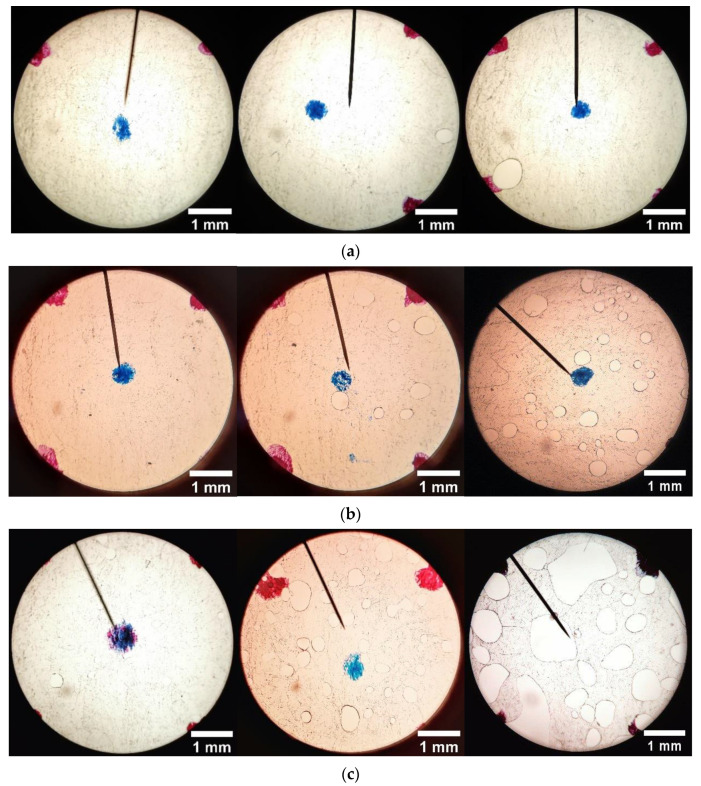
Typical microscopic images (40×) for (**a**) 16.2 kGy; (**b**) 56.2 kGy; (**c**) 114.4 kGy. Hot press conditions: 6228 N, 216 °C, 2 min.

Due to time constraints, 3 irradiation dose levels were inspected for porosity for each dose range; two wafer samples were measured for at each selected dose. The microscope images were used to generate plots of area-averaged porosity as well as to gauge the relative sizes with increasing dose–typical images at three grid locations for 3 dose levels in each of the two dose ranges, as shown in Figure 20 and Figure 21, respectively. 

It can be observed that the pore distribution was not uniform for each individual sample. On the same sample, there could be areas where little or no pores are present, and areas where large portions of pores exist as well. 

**Figure 22:**
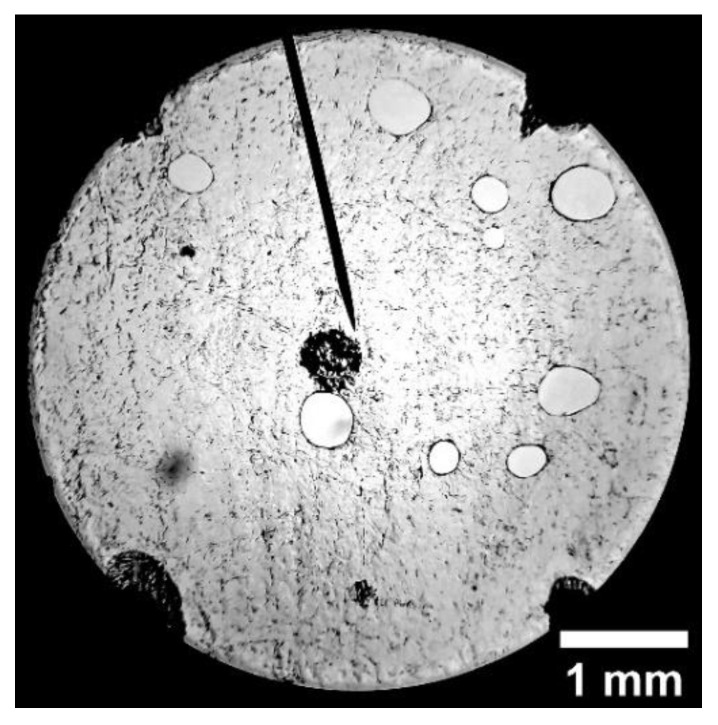
Example of a processed image for a 56 kGy sample.

To count the pores more precisely, the images were processed and sharpened, as illustrated in Figure 22. The dimensions were scaled using an AmScope^TM^ Microscope Stage Calibration Slide. To eliminate the uncertainty brought about by the external camera, the areas measured on each microscopic image were also normalized with the total area within the scope.

Porosity (*P*), or pore fraction, was determined by adding the total pore area of all pores taken on each sample and dividing the total area of all pictures within the scope of the microscope:

(2) $$P=\frac{\mathrm{Total}\;\mathrm{pore}\;\mathrm{area}\;\mathrm{within}\;\mathrm{all}\;\mathrm{grids}\;\mathrm{selected}}{\mathrm{Total}\;\mathrm{area}\;\mathrm{of}\;\mathrm{all}\;\mathrm{selected}\;\mathrm{grids}\;}$$

**Figure 23:**
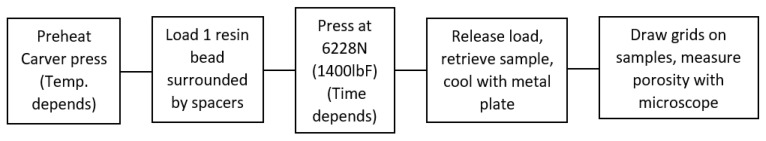
Steps in porosity-related wafer sample production and estimation.

Figure 23 presents a flow-chart summary of the steps undertaken.

Porosity-related testing was conducted for the test parameters summarized in Table 6.

## 4. Results and Discussion

This section separately presents and discusses results from experiments for the MLR and porosity metrics for gamma irradiation dose ranges. 

### 4.1. Results of the MLR Experiments

**Figure 24:**
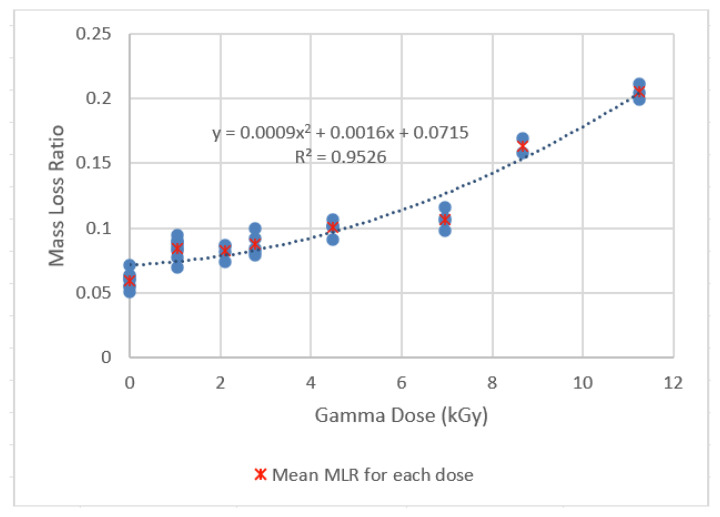
MLR of the PLA resin irradiated over 0–11 kGy gamma dose (Hot Press Conditions: 227 °C, 16 min hold time).

**Figure 25:**
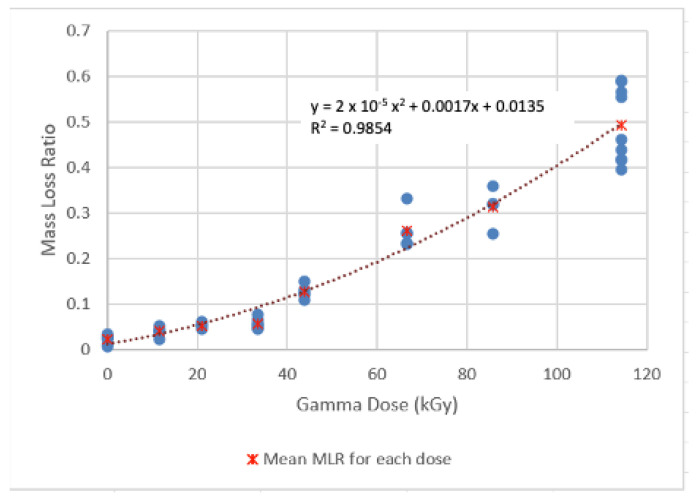
MLR of the PLA resins irradiated over 11–120 kGy gamma dose (Hot Press Conditions: 193 °C, 12 min hold time).

The MLR of hot pressed PLA resin for low dose range (0–11 kGy) and higher dose range (11–120 kGy) are shown in Figure 24 and Figure 25. The statistical analysis of the results is shown in Table 7 and Table 8, respectively. Correlations using the least-squares approach for estimating MLR over the two specific Dg ranges examined were developed and are presented below:

MLR = 0.0009 Dg^2^ +0.0016 Dg + 0.0715          R^2^ = 0.9526; 0 < Dg < 11 kGy (3)

MLR = 2 × 10^−5^ Dg^2^ +0.0017 Dg + 0.0135          R^2^ = 0.9854; 0 < Dg < 120 kGy (4)

#### Heuristic Mathematical Model for Rheology Based MLR Metric

To depict the mass loss model, the relationship between MLR and the applied gamma dose was determined by fitting the response curve. On this basis, the well-known volumetric thermal expansion theory was adapted as:

∆V = V_0_ β (T_1_ − T_0_) (5)

where, ∆V is the volume change of an object when the temperature rises from T_0_ to T_1_, V_0_ is the volume at T_0_, β is the volumetric coefficient of expansion, and β = 3α for a rectangular body, where α is the linear coefficient of thermal expansion for the material.

Coefficient of thermal expansion (CTE) α is therefore calculated for each sample with the following relationships:

∆V = m_r/_ρ (6)

V_o_ = m_0_/ρ (7)

where, m_o_ is the original mass, m_r_ is the lost mass, ρ is the density and V_o_ is the volume of PLA, respectively. We simplify the rheology treatment of molten PLA leaking from the mold under certain temperature/time conditions to a thermal expansion model of a solid PLA chip and treat the mass lost (m_r_) as the extruded mass from the mold assuming constant density. A “pseudo coefficient of thermal expansion” α_ps_ is derived in relation to the mathematical model for MLR:

(8) $$\mathrm{MLR}=\frac{m_{r}}{m_{0}}=\frac{\Delta V*\rho }{m_{0}}=\frac{3V_{0}{\;\alpha }_{\mathrm{ps}}\;\left(T_{1}-{\;T}_{0}\right)*\rho \;}{m_{0}}=A*\;\left(T_{1}-{\;T}_{0}\right)$$

where, $A=\frac{3V_{0}{\;\alpha }_{\mathrm{ps}}\;\rho \;}{m_{0}}=3\alpha_{\mathrm{ps}}\;$is a constant, indicating MLR is linearly related to the temperature change. Combining Equations (3) and (4), it can be found that for samples prepared at a certain temperature,

α_ps_ = (0.0009 Dg^2^ + 0.0016 Dg + 0.0715)/3          0 < Dg < 11 kGy (9)

α_ps_ = (2×10^−5^ Dg^2^ + 0.0017 Dg + 0.0135)/3          0 < Dg < 120 kGy (10)

As aforementioned, pores are generated inside molten PLA upon heating, which drove the excessive PLA melt to escape from the mold and caused mass loss. This “pseudo CTE” measures the amount of molten PLA excluded from the mold upon heating in a confined volume, which in reality is a coefficient correlated to porosity. In other words, the more pores are generated, the larger the “pseudo CTE,” the more material is lost. Given the fact that irradiation causes little change in the density of PLA resin, there should be a linear relationship between porosity and mass loss. 

This assumption seems to agree well with the experimental results, wherein a quadratic relationship is found in both curves (discussed in the next subsection). In theory, if porosity is accurately measured, the following equation would hold:

(V_0_ ∗ P) ∗ ρ = m_r_ (11)

where, V_0_ is the volume of the the mold cavity (assuming no distortion), P is the porosity, ρ is the density of PLA, and, m_r_ is the mass removed (i.e., loss).

Considering V_0_ ∗ ρ = m_0_ and MLR = m_r_/m_0_, Equation (11) is reduced to,

P = MLR(12)

In Section 4.2, this derivation will be examined for validity. 

### 4.2. Results of PLA Porosity Metrics for Gamma Dosimetry

**Figure 26:**
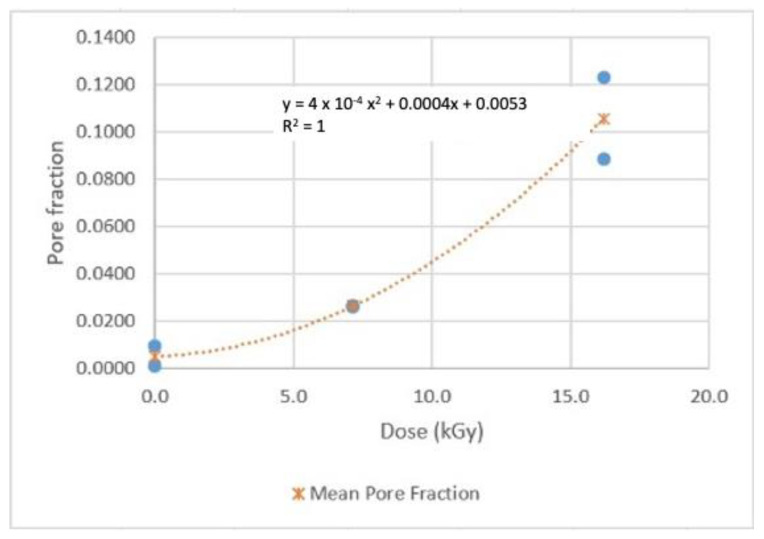
Porosity (pore fraction) of the 0–16 kGy wafer samples.

**Figure 27:**
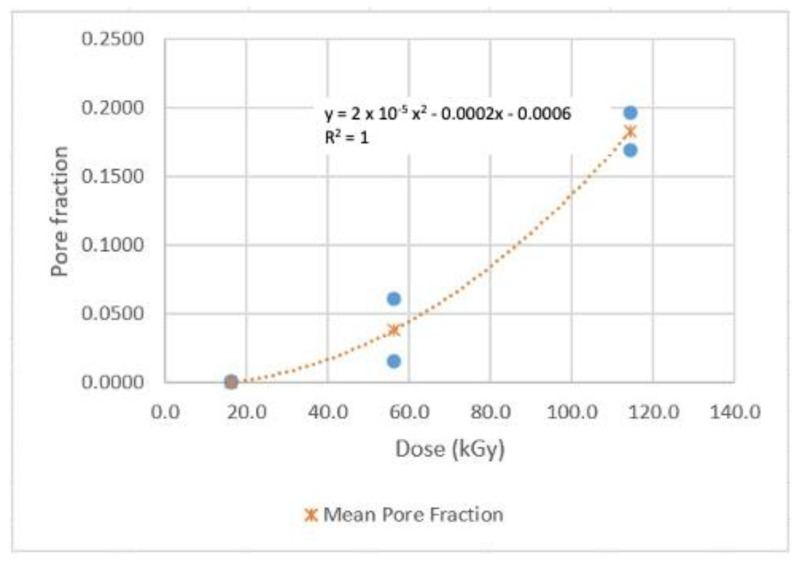
Porosity (pore fraction) of the 16–114 kGy wafer samples.

As stated in Section 3.6, two samples were measured for each dose. The average porosity of the two was calculated, and the results are shown graphically in Figure 26 and Figure 27. Correlations using the least-squares approach for estimating pore fraction (P) over the two specific Dg ranges examined were developed and are presented below:

P = 0.0004 Dg^2^ + 0.0004 Dg + 0.0053          R^2^ = 1; 0 < Dg < 16 kGy (13)

P = 2 × 10^−5^ Dg^2^ − 0.0002 Dg − 0.0006          R^2^ = 1; 16 < Dg < 114 kGy (14)

It’s clear that the pore fractions increase as the dose increases quadratically in both dose ranges. The average pore fraction reached is ~11% for the 0–16 kGy range, and ~18% for the 16–114 kGy range, as noted from Figure 26 and Figure 27, respectively. Notably, the pore fractions measured for two random samples for each dose were reasonably close to each other. Despite the limited number of tests and uncertainties, it is safe to say that porosity may be another viable metric for PLA dosimetry. 

Looking back to the discussion in Section 4.1, unfortunately, the porosity–dose correlation cannot be directly related to the MLR–dose correlation, since the sample preparation protocols were different. Regardless, when comparing Equations (3) and (4) and Equations (13) and (14), it is noticed that the equations for the low dose range (<20 kGy) and the equations for the high dose range (20–120 kGy) for both methods have similar forms–the coefficients for the highest order in the low dose range equations are both in the 10^−4^ order while which in high dose range equations are both in the 10^−5^ order. This indicates an inherent correlation and inter-relationship between porosity and the mass loss ratio. 

## 5. Summary and Conclusions

As a “green,” renewable corn-soy based polymer, PLA has promising potential to be developed into a cheap and efficient dose indicator, taking advantage of its degradation effect upon irradiation. In this paper, two novel approaches to PLA dosimetry are presented. The first (MLR) approach is based on rheology, and the second (Porosity Fraction) is based on induced porosity levels post-irradiation.

MLR constitutes an approach reflecting the mobility of the melt leaking out from a half-sealed mold. The gamma response of PLA was investigated via MLR in this study. Different temperature/time combinations were adopted to check the MLR at different dose ranges. Neat response curves were found in both ranges.

As an extension study of the MLR metric, the porosity of PLA resins was developed into another metric for PLAD-based gamma dosimetry. A separate testing matrix was determined for porosity measurement, and similar to MLA, different conditions were applied to different dose ranges. These results were compared with those of MLR studies. While limited similarity was found, more needs to be done to better quantify the inter-relationships between the two metrics. In this connection, as well, to improve upon and automate the processing of pore image scans for rapidly deriving the porosity metric.

The PLAD technology discussed in this paper utilized semi-crystalline form PLA, for which the crystalline nature of the polymer with irradiation could well be affected during the cooling phase post-compression at elevated temperatures. As such, morphological changes pertaining to crystallinity may also be useful for further characterizing gamma dosimetry, e.g., via wide angle x-ray diffraction (WAXD) techniques. 

As mentioned earlier in the Introduction section, gamma radiation detection is a well-established field [1,2,3,4]. PLAD may also be of utility for space-based applications where, at present, various detector types such as InGaAsP/InP resonators, and Si-on-Si and Si-on-insulator microphotonic devices are being researched [26,27] together with PLA. Ref. [28] studies examined gamma irradiated 3-D printed PLA samples for morphological changes using well-established laboratory techniques such as FTIR, DSC and structural-impact strength related properties (tensile/bending, elongation, modulus of rupture, hardness, etc.) of specimens. The techniques used for this study showed little to no significant changes for Co-60 gamma doses below 50 kGy. 

Overall, the PLAD’s MLR approach represents a novel, effective and simple approach for enabling PLA resin to be used for medical gamma dosimetry for the interesting (biomedical field relevant) dose range spanning 1–100 kGy. So far, only the γ response of PLAD has been studied; it is expected that this approach can be applied to other types of ionizing irradiation as well, i.e., electron, neutron and alpha irradiation. 

## Figures and Tables

**Table 1:** Physical properties for Ingeo™ Biopolymer 4043D [20,21].

Parameter	Value
Specific Gravity, g/cc	1.24
Relative Viscosity	4.0
Melt Temperature, °C	145–160
Glass Transition Temperature, °C	55–60
Decomposition temperature, °C [21]	250
Mass Flow Rate, g/10 min, for 210 °C/2.16 kg conditions	6

**Table 2:** Mass loss ratio (MLR) for 124 kGy resin tested at 177 °C for 20 min.

Dose (kGy)	MLR
0	0.0225
124	0.1250

**Table 3:** Testing matrix (Loading Force: 6672 N (1500 lbf)).

Doses Tested (kGy) (Resins Irradiated in 2021)	Temperature (°F/°C)	Hold Time (min)	Rest Time (min)
0, 1.0, 2.1, 2.8, 4.5, 7.0, 8.7, 11.2	440/227	16	1
0, 11.4, 21.0, 33.4, 43.8, 66.7, 85.8, 114.4	380/193	12	1

**Table 4:** Density of PLA 4043D resins irradiated with 0, 66.7 and 114.4 kGy gamma doses.

Gamma Dose (kGy)	Mass (g)	Volume (mL)	Density (g/mL)
0	5.0	4.2	1.19
66.7	5.0	4.0	1.25
114.4	5.0	4.2	1.19

**Table 5:** Hot press test parameters for porosity approach.

Dose Range (kGy)	Temperature-°C (F)	Hold Time (min)	Force-N (lbf)
0–16	232 (450)	5	6228 (1400)
16–115	216 (420)	2	6228 (1400)

**Table 6:** Experiment test matrix for porosity metric.

Doses Tested (kGy)	Temperature (°F/°C)	Hold Time (min)
0, 7.1, 16.2	450/232	5
16.2, 56.2, 114.4	420/216	2
Hot Pressed Under 6228 N (1400 lbf)		

**Table 7:** Statistical analysis of the MLR of the PLA resins irradiated with a 0–11 kGy gamma dose.

Dose (kGy)	Mean	1 σ	Median	Max.	Min.
0	0.0596	0.0064	0.0600	0.0708	0.0502
1.0	0.0843	0.0079	0.0857	0.1016	0.0914
2.1	0.0820	0.0057	0.0836	0.0867	0.0741
2.8	0.0872	0.0087	0.0841	0.0998	0.0788
4.5	0.1002	0.0054	0.1012	0.1060	0.0914
7.0	0.1065	0.0072	0.1064	0.1155	0.0979
8.7	0.1635	0.0083	0.1635	0.1694	0.1577
11.2	0.2052	0.0061	0.2047	0.2115	0.1995

**Table 8:** Statistical analysis of the MLR of the PLA resins irradiated with an 11–120 kGy gamma dose.

Dose (kGy)	Mean	1 σ	Median	Max.	Min.
0	0.0240	0.0093	0.0266	0.0348	0.0082
11.4	0.0407	0.0097	0.0420	0.0541	0.0240
21.0	0.0537	0.0064	0.0534	0.0618	0.0463
33.4	0.0586	0.0113	0.0562	0.0775	0.0475
43.8	0.1278	0.0154	0.1250	0.1514	0.1097
66.7	0.2618	0.0405	0.2539	0.3316	0.2328
85.8	0.3134	0.0432	0.3202	0.3587	0.2547
114.4	0.4922	0.0813	0.4610	0.5908	0.3954

## Data Availability

Upon request from corresponding author.

## Abbreviations

Acronyms Used: PLA: Polylactic acid; Dg: Gamma dose; ML: Mass loss; MLR: Mass loss ratio.
